# Modification of Polycaprolactone with Plant Extracts to Improve the Aging Resistance

**DOI:** 10.3390/ma16145154

**Published:** 2023-07-21

**Authors:** Krzysztof Moraczewski, Magdalena Stepczyńska, Rafał Malinowski, Tomasz Karasiewicz, Bartłomiej Jagodziński, Piotr Rytlewski

**Affiliations:** 1 Faculty of Materials Engineering, Kazimierz Wielki University, Chodkiewicza 30, 85-064 Bydgoszcz, Poland; m.stepczynska@ukw.edu.pl (M.S.); tomakara@ukw.edu.pl (T.K.); bar.jag@ukw.edu.pl (B.J.); prytlewski@ukw.edu.pl (P.R.); 2 Łukasiewicz Research Network—Institute for Engineering of Polymer Materials and Dyes, Marii Skłodowskiej-Curie 55, 87-100 Toruń, Poland; malinowskirafal@gmail.com

**Keywords:** polycaprolactone, polyphenols, plant extracts, accelerated aging

## Abstract

Natural extracts of plant origin are used as anti-aging compounds of biodegradable polymers. Coffee, cocoa, or cinnamon extracts in amounts from 0.5 to 10 wt.% were added to the polycaprolactone matrix. The manufactured materials were aged at elevated temperatures with increased relative humidity and continuous exposure to UV radiation for 720, 1440, or 2160 h. The performance of the proposed extracts was compared with the retail anti-aging compound, butylated hydroxytoluene. Visual assessment, FTIR analysis, melt flow rate, tensile strength, impact tensile strength, thermogravimetry, and differential scanning calorimetry tests were conducted. Results showed that the use of lower contents of the tested extracts is particularly advantageous. When the content of the extract did not exceed 1 wt.%, no unfavorable influence on the properties of the materials was observed. The stabilizing performance during accelerated aging was mostly similar to or greater than that of the reference compound used.

## 1. Introduction

As materials and products are utilized, they undergo aging which can lead to a decline in their visual and functional characteristics [1]. Due to the prevalence of this phenomenon, multiple research centers worldwide are dedicated to conducting studies aimed at mitigating its negative impact [2,3,4]. Currently, the demand for polymeric materials is on the rise, leading to extensive research on the aging mechanisms of these materials. The research focuses on understanding the complex aging processes and their effects on the physical and chemical properties of polymeric materials, as well as the influence of processing on said aging phenomena [5,6,7,8].

Extensive research has been conducted over the years to enhance the resistance of polymers to atmospheric, chemical, and thermal factors. Anti-aging compounds have proven to be the most effective approach to improving the stability of polymer materials. These compounds, whether natural or synthetic, are designed to increase the resilience of polymer materials against external factors such as high temperatures, UV radiation, and chemicals [9,10,11,12].

As the push for more durable polymers continues, a growing global focus on ecological sustainability has also continued. The primary objective is to find effective solutions to protect the environment. One promising approach is promoting and implementing biodegradable polymers across various industries and applications.

The biodegradable polymer is completely transformed by microorganisms (bacteria and fungi) into carbon dioxide, water, and humus [13] and can be successfully processed using most standard polymer processing technologies, including the most popular: thermoforming, extrusion, injection, or blow molding [14,15,16,17,18].

As of late, there has been an increasing demand for polymer materials that can withstand external factors while being entirely biodegradable at the end of their lifespan. To achieve this, one must select a suitable biodegradable polymer/anti-aging compound combination.

One of the widely used biodegradable polymers is polycaprolactone (PCL) [19,20,21,22,23,24,25]. PCL is an aliphatic polyester, obtained from caprolactone by ring-opening polymerization. It is easily mixed with many other polymers, therefore it is very often used as a plasticizer increasing the flexibility of polymers, as well as an additive increasing biodegradability. In combination with starch, it is used to make rigid plastic from which disposable plates or cups are made, which can be disposed of by composting. This polymer has found many biomedical applications. It is commonly used in the production of implants and absorbable surgical threads due to the gradual and slow decomposition caused by the hydrolysis of ester bonds in the human body, which takes approximately two years [26,27,28,29].

When it comes to using anti-aging compounds for biodegradable polymers, certain limitations must be considered. These compounds must not disrupt the composting process and should be non-toxic, non-volatile, and safe for contact with food, as the packaging industry is a major user of biodegradable polymers, particularly for food packaging. It would be highly beneficial to opt for plant-based products, as they decompose easily and do not impede the composting process. Additionally, plant-based substances are environmentally friendly. Substances containing natural polyphenols derived from plants show enormous potential as effective anti-aging compounds due to their composition and chemical structure. Polyphenols are readily available and do not require special chemical synthesis processes [30,31].

Natural antioxidants are classified into various groups, with polyphenols being the largest among them. They exhibit a wide range of molecular weights, structures, and biological and physicochemical properties. Due to the diverse nature of polyphenols in terms of structures and properties, it can be challenging to systematize them. Phenolic compounds can be very generally divided into four general groups: phenolic acids, phenolic diterpene, flavonoids, and volatile oils [32]. They are secondary metabolites widespread in the plant world (usually in the form of glycosides or esters) but not found in animals. They are found in fruits, vegetables, plant-based beverages, spices, and herbs. Plant polyphenols can act in several of the following ways: reducing agents, free radical blockers, metal ion chelating agents that catalyze oxidative reactions, and agents preventing reactions caused by a single active oxygen atom [33,34,35,36,37].

Natural polyphenols are becoming a popular ecological substitute for synthetic anti-aging compounds due to their versatility. The literature offers insights into how different polyphenols can stabilize various polymer types. One can find the use of such polyphenols as gallic, vanillic, caffeic, cinnamic, sinapic, syringic, ferulic, coumaric, and ascorbic acids, tocopherols, quercetin to stabilize polyethylene, polypropylenes, starch, cellulose, polylactide, polyhydroxyalkanoates, and poly (ethylene oxide) [38,39,40,41,42,43].

The main objective of the conducted study was to determine whether natural plant-derived substances containing polyphenols could potentially serve as anti-aging compounds for polycaprolactone. The proposed approach will improve the resistance of biodegradable polycaprolactone to external factors without negatively affecting biodegradation during composting. The results of selected properties of unaged and aged PCL containing from 0.5 to 10 wt.% of coffee, cocoa, or cinnamon extract are presented. Detailed studies of the influence of the proposed extracts on selected properties of polycaprolactone have already been carried out and published [44]. In this paper, selected properties of PCL containing natural anti-aging compounds subjected to the accelerated aging process were determined and compared to properties of materials containing a commercially used anti-aging compound.

## 2. Materials and Methods

The materials matrix used was polycaprolactone (PCL) type Capa 6800 from Perstorp, Sweden. It had a mass melt flow rate (MFR) of 2–4 g/10 min (160 °C, 5 kg), density (ρ) of 1.10 g/dm^3^, mean molecular weight of 80,000, melting point 58–60 °C, and glass transition temperature of −60 °C. To enhance its properties, 3 natural compounds of plant origin were added: coffee (*Coffea arabica* L.) extract, cocoa (*Theobroma cacao* L.) extract, and cinnamon (*Cinnamomum cassia* Presl) extract, sourced from Agrema Sp. z o.o., Wrocław, Poland. The coffee extract contained 45 wt.% of polyphenols, while cocoa and cinnamon extracts had 5 wt.% of polyphenols. Chlorogenic acid, flavonoids, and phenolic acids were the main polyphenols in coffee, cocoa, and cinnamon extracts, respectively.

To assess the quality of the acquired materials, they were compared to a commonly used synthetic anti-aging compound. Butylated hydroxytoluene (BHT) from Sigma-Aldrich, St. Louis, MO, USA, was used as the reference compound, with a density of 1.04 g/dm^3^. BHT is a popular anti-aging additive for multiple polymers [45,46,47].

Initially, the test materials were created through an extrusion process. The granules of the PCL composites under examination were extruded with the aid of a co-rotating twin screw extruder BTSK 20/40D (Bühler, Germany). The parameters of each extruder zones were 115, 115, 120, and 120 with a head temperature of 120 °C. 0.5; 1; 3; 5 or 10 wt.% of the proposed natural anti-aging extract was added to the polymer matrix, giving samples marked as P0.5, P1, P3, P5, and P10, respectively. A sample of pure PCL was designated as P. Usually, the content of traditional anti-aging compounds typically used in the processing and use of polymeric materials does not exceed a few percent. In the conducted research, in addition to the small contents of the extracts, larger amounts of these materials were used. Unlike synthetic anti-aging compounds, the active substance is only a part of the weight of the extract added to the polymer; therefore, it seems advisable to use higher concentrations of extracts. As a reference sample, a material containing 2 wt.% BHT was also extruded. The reference material was designated as the R sample.

During the second phase of processing, the test samples were created. Using a Plasti-Corder Lab Station (from Brabender, Germany), composite films were extruded with a single-screw extruder that had a flat-faced head with a mouthpiece width of 170 mm. The cylinder zones were heated to temperatures of 75, 85, and 95 °C, while the head temperature was at 100 °C. Afterwards, we placed the melted polymer onto cold thermostatic rolls that were at around 20 °C. The resulting film had a thickness of roughly 0.5 mm.

The accelerated aging process was carried out using a CCK 40/300NG (Dycometal, Barcelona, Spain) climatic chamber. The aging process was performed in a closed chamber at a temperature of 40 °C, 70% relative humidity, and UV irradiation for 720, 1440, or 2160 h. The apparatus was equipped with 8 UV lamps (PHILIPS SUPER ACTINIC TL 60 W/10-R ISL, Philips, Amsterdam, The Netherlands) with a wavelength range of 350–400 nm with a peak intensity of 370 nm. The sample/lamp distance was kept as small as possible by placing the samples directly under the UV lamps. The distance between the lamps and the surface of the samples was approx. 10 cm. The distance used was selected based on the experience of the operator of the climatic chamber. A shorter distance would cause the surface temperature of the samples to be too high, which could melt them. If the lamps were too far apart, the intensity of UV radiation could be too low to effectively age the material.

Color tests were carried out on films with a thickness of 500 μm using a BYK colorimeter (BYK-Gardner GmbH, Geretsried, Germany) designed for measuring the color and gloss of materials. The color measurement was quantified using a color space standardized by the CIELab system based on 3 components: L, a, and b. The component “L” describes the lightness of the material, the component “a” describes the color in the range from green to red, and the component “b” describes the color in the range from blue to yellow. The color difference ΔE and browning index (BI) [48] were calculated according to the equations:

(1) $$∆E=\sqrt{{∆L}^{2}+{∆a}^{2}+{∆b}^{2}}$$

(2) $$\mathrm{BI}=\frac{100\left[\left(\frac{a+1.75L}{5.645L+a-0.3012b}\right)-0.31\right]}{0.17}$$

where ΔL is the difference in L between 2 samples (virgin PLA and PLA with specific extract content) while, similarly, Δa and Δb are the differences in the a and b coordinates, respectively.

For samples without the extracts, instead of the browning index, the yellowness index (YI) was calculated according to the YID1925 standard from Equation (3) by converting the L, a, and b color scale into the XYZ color scale.

(3) $$\mathrm{YI}=100\left(1-\frac{0.847Z}{Y}\right)$$

An FTIR Nicolet iS10 spectrophotometer (ThermoScientific, Waltham, MA, USA) was used to record sample spectra. The attenuated total reflection method (ATR-FTIR) was used to obtain Fourier transform infrared spectra (FTIR). The analyzed spectrum was an average of 16 measurements taken between the wave number range of 4000 to 650 cm^−1^.

An MP 600 plastometer (Tinius Olsen, Horsham, PA, USA) was used in the melt flow rate (MFR) tests. The MFR (100 °C, 5 kg) was determined using 12 individual samples. The final values of MFR were calculated as the arithmetic mean of 10 results (2 extreme values were neglected).

An Instron 3367 (Instron, Norwood, MA, USA) tensile machine was used in mechanical properties tests. The extension rate for testing each sample was 200 mm/min. Tensile tests were carried out on standardized samples in the formation of paddles with measuring section of 100 × 10 × 4 mm. Mechanical parameters were determined using 12 individual samples. The final values were calculated as the arithmetic means of 10 results, with 2 extreme ones being neglected.

An IMPASS-15 (ATS FAAR, Cassina dè Pecchi, Italy) impact hammer was used in impact tests. A 4J hammer was utilized with a speed of 2.9 m/s to break samples in the form of 80 × 10 × 4 mm bars. The impact strength was determined through 12 individual samples, and the final values were calculated as the arithmetic means of 10 results after disregarding 2 extreme values.

A Q500 differential scanning calorimeter (TA Instruments, New Castle, DE, USA) was used in thermal properties tests. The experiments were conducted under a nitrogen atmosphere, utilizing samples weighing approximately 7 mg and a temperature range from −70 to 80 °C. The data were captured in 3 separate cycles, which included first heating, cooling, and a second heating process with a heating rate of 10 °C/min.

A Q200 thermogravimetric analyzer (TA Instruments, USA) was used in the thermal stability tests. The experiments were performed under a nitrogen atmosphere. We utilized samples that weighed around 22.5 mg and conducted the tests within a temperature range from 20 to 600 °C while heating them.

## 3. Results and Discussion

### 3.1. Visual Evaluation

**Figure 1:**
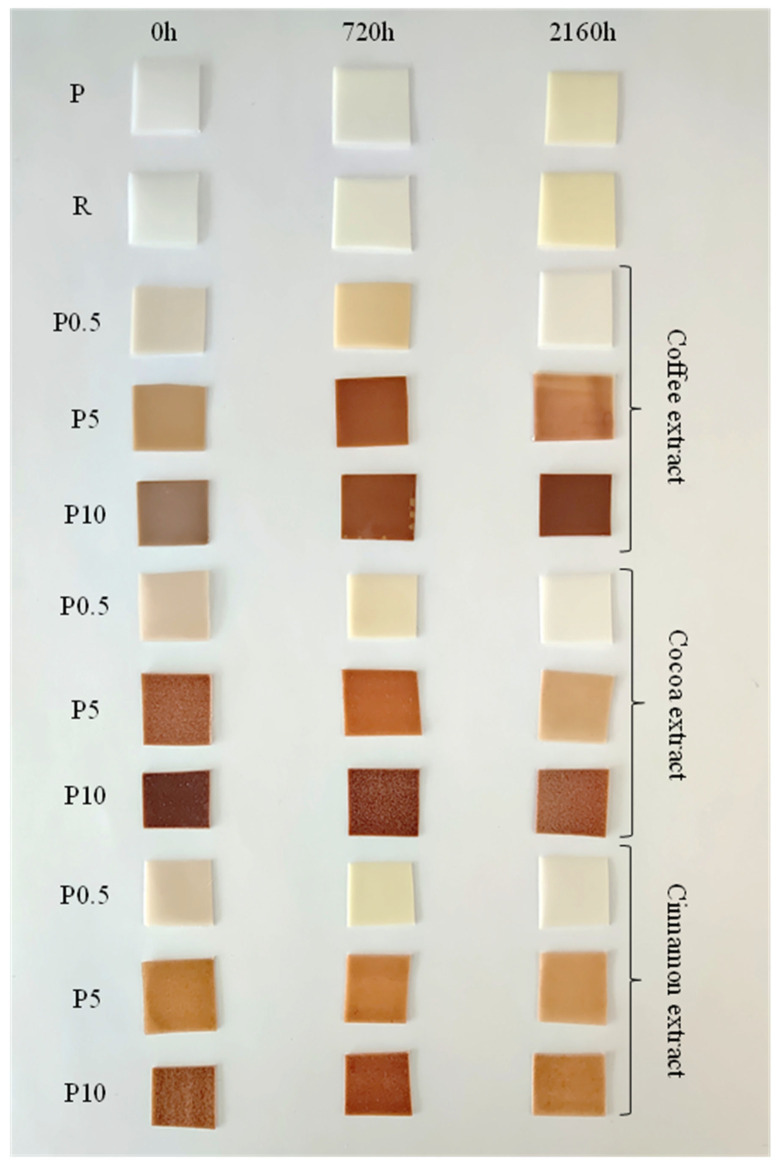
Photos of selected samples before and after the aging process. Approximate sample size 10 × 10 mm.

For the polymeric components and parts used in indoor and outdoor applications, changes in color because of aging phenomena could be an important problem and challenge. The color differences of the samples before and after aging were assessed by comparing their photographic images. Pictures of selected un-aged samples and samples aged for 720 or 2160 h are shown in Figure 1.

To support the visual assessment with experimental data, the color tests in the CIElab color space were carried out and some of the parameters are presented in Table 1.

The color of samples P and R were identical. The ΔE value of sample R was 0.64. According to the CIELab color space rules, when ΔE < 1, the standard observer will not notice the difference between the two colors [43,49]. The introduction of the tested extracts to the PCL matrix resulted in clear changes in the color of the material. The samples turn from non-transparent white into brown, the shade of which depends on the type of extract and the intensity of the extract content. The value of the L component, which indicates the brightness of the samples, decreased with the increase in the extract content, with the simultaneous increase in the ΔE parameter, which for all samples had values above five, i.e., the color change was easily noticeable with a distinct impression of two different colors. The more extract in the material, the more intense the brown was, which was confirmed by the growing BI values.

Due to the accelerated aging process, as an effect of UV radiation, the color of the samples changed. The first easily noticeable effect of polymer aging is the yellowing of the material. It is caused by several factors, including UV radiation, heat, or liquids. The effect of UV radiation on matter is related to the processes of photolysis, i.e., the cleavage (breaking) of chemical bonds due to radiation absorption. This process breaks chemical bonds and creates two compounds each with one unpaired electron, which stimulates the formation of very reactive free radicals. They react with other compounds, creating a new compound and another free radical. The reason for the color change (yellowing) is precisely the formation of these radicals (because these new molecules reflect some light, in many cases yellow). Such a yellowing effect was observed in the case of the pure PCL sample (sample P), where after 2160 h of aging the material had a yellow shade. A numerical representation of the yellowness effect is a substantial change in the calculated YI value from 3.37 for unaged PCL to 27.79 for PCL aged for 2160 h. Interestingly, the unfavorable color change was not stopped by the addition of the reference anti-aging compound (BHT) because sample R also turned yellow. The calculations showed that the yellowing effect was even more intense than pure PCL, as the YI value increased from 0.64 for the unaged R sample to 32.61 for the R sample aged 2160 h.

In the case of samples containing the tested extracts, accelerated aging caused a decrease in color intensity, which was caused by the influence of UV radiation on the chromophore groups. This effect was most visible at low extract contents, where after 2160 h of aging the color practically disappeared completely. The visual observations were confirmed by the values of the L parameters obtained with the use of a colorimeter and the calculated values of ΔE and BI. The L component in most cases increased with the aging time, confirming the increase in brightness of the samples. The decrease in the intensity of the brown color visible in the photos was confirmed by the lower BI value of individual samples with increasing aging time, as well as a decrease in the ΔE parameter. Due to the high energy, ultraviolet radiation can cause photochemical reactions in the illuminated object. Chemical decomposition is also subject to the chemical decomposition of compounds giving color to the tested materials. This process is stronger, resulting in the darker colors of the samples, which is confirmed by the presented results. The exceptions were samples containing coffee extract, in the case of which the shortest aging period was followed by a remarkably interesting effect of “enhancing” the color, visible in the photos and supported by numerical results.

Color change from colorless to brown should not be a problem in the possible use of tested material films. They can be successfully used in applications where color is not of great importance, e.g., disposable dishes, straws, general medical applications, or garbage bags. The biggest advantage of these products will be their biodegradability and lower environmental burden, which may be more important for the potential consumer.

### 3.2. Spectroscopic Studies

The analysis of the spectrum of pure PCL and the materials containing the tested extracts (not shown here) showed the occurrence of absorption bands typical of this polymer. The introduction of extracts into the matrix did not cause any significant changes in the spectrum. Only the appearance of a small band in the range of 3600–3200 cm^−1^ was observed, the intensity of which increased with the amount of extract in the material. This band originates from the -OH groups of polyphenols contained in the extracts. The lack of significant changes in the image of the spectra suggests that the added extracts tend to accumulate in the core of the material and not in its surface layer.

In the FTIR analysis, detailed attention was focused on the 1800–1650 and 1500–1000 cm^−1^ regions allowing the assessment of the degree of oxidation of the surface layer. The 1720 cm^−1^ peak is from stretching vibrations of carbonyl group C=O, the 1420 cm^−1^ peak is from deformation vibrations of CH_2_–C=O, the 1294 cm^−1^ peak is from C–O stretch in the crystalline phase, the 1242 cm^−1^ peak is from asymmetric COC stretching, and the 1189 cm^−1^ peak is from OC-O stretching.

**Figure 2:**
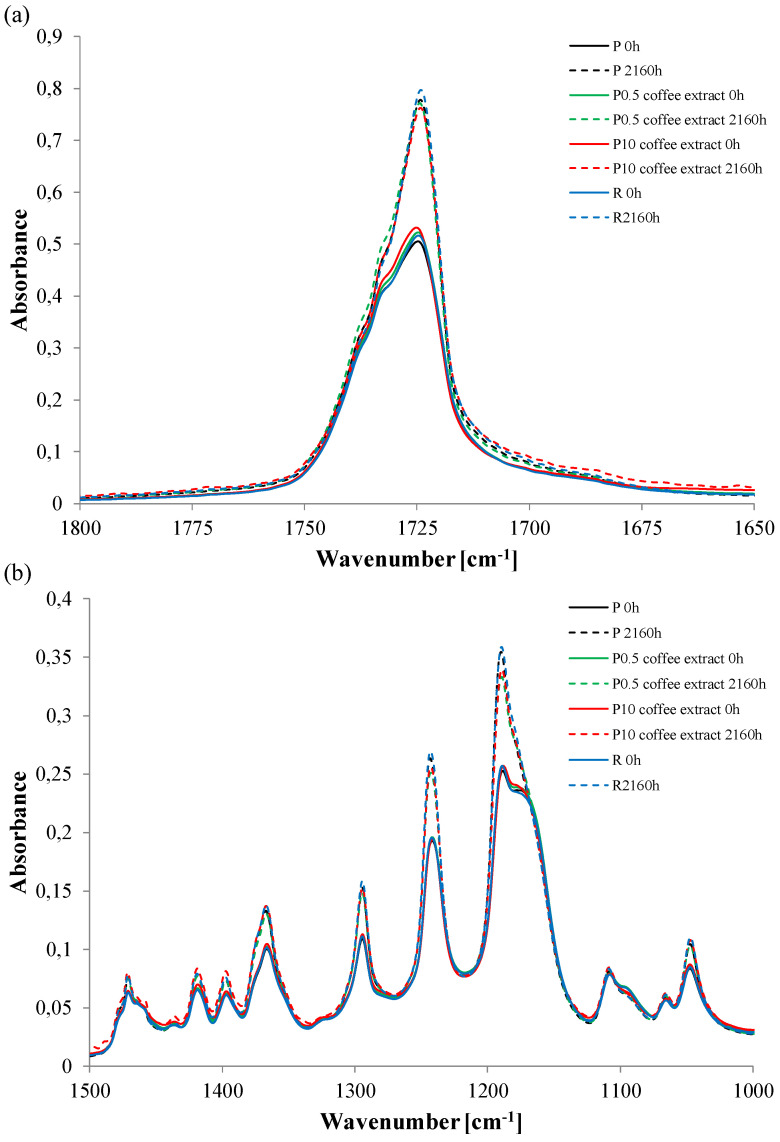
FTIR spectra of (**a**) 1800–1650 cm^−1^ and (**b**) 1500–1000 cm^−1^ regions of selected samples.

Two groups of spectra are visible in Figure 2. Spectra of unaged samples (solid line) with lower peak intensities and spectra of aged samples (dashed line) with higher peak intensities. Thus, it can be seen that because of accelerated aging (mainly because of UV radiation), there was a significant oxidation of the surface layer of the tested samples. The lack of differences between the samples without and with the extracts indicates that unfortunately, the addition of extracts did not reduce the oxidation of the polymer surface. The applied extracts, however, were slightly more effective than the reference anti-aging compound, which, interestingly, also did not limit oxidation.

### 3.3. Melt Flow Rate

The MFR value for sample P, which was pure PCL, was 1.65 g/10 min. Upon adding the reference anti-aging compound (sample R), the MFR increased slightly to 1.89 g/10 min. The addition of tested extracts to the polymer matrix also resulted in changes in MFR. The MFR result for all samples is presented in Table 2.

Comparing the MFR of pure PCL to the MFR of PCL mixed with coffee extract, the latter showed an increase from 0.11 g/10 min for 0.5 wt.% to 0.31 g/10 min for 10 wt.%. Similarly, adding 0.5 wt.% of cocoa extract resulted in a significant increase in MFR by 0.43 g/10 min compared to the value of sample P. However, unlike the coffee extract, increasing the amount of cocoa extract decreased the MFR, with a difference of only 0.06 g/10 min for the sample with 10 wt.%. As for cinnamon extract, after an initial increase of 0.14 g/10 min at 0.5 wt.%, the MFR value decreased. The lowest MFR value was obtained for the sample containing 3 wt.% of cinnamon extract, with a subsequent increase in extract content leading to an increase in MFR. The highest MFR value was obtained for the sample containing 10 wt.% of the extract, which was higher by 0.29 g/10 min than the pure PCL value.

The increase in MFR after the addition of the extracts was caused by the migration of short polyphenol molecules between the PCL polymer chains. This caused an increase in the distance between the macromolecules, weakening their mutual forces, thus facilitating a chain slip under the pressure increase in the flow of the polymer mass [50].

**Figure 3:**
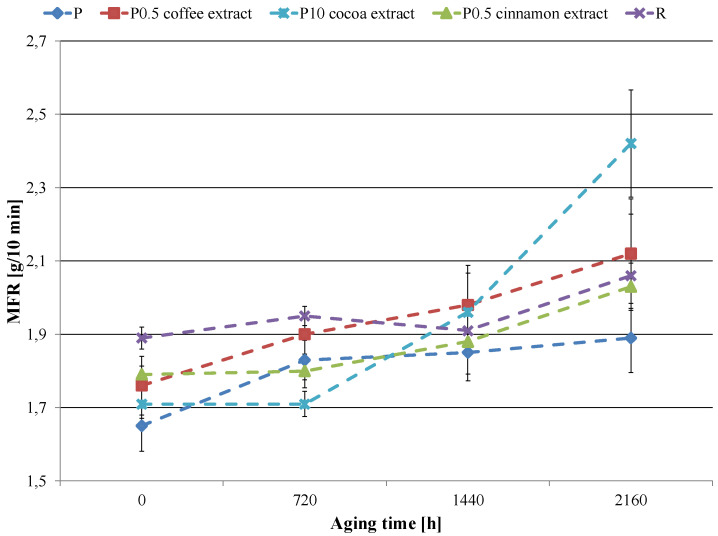
Melt flow rate (MFR) values of selected samples as a function of aging time.

Changes in the MFR value as a function of aging time for selected samples are shown in Figure 3. Aging the P sample increased the MFR of pure PCL by 0.2 g/10 min compared to the unaged sample. The use of the reference anti-aging compound slightly limited this increase, as the MFR value of the R sample aged for 2160 h increased by 0.17 g/10 min. Changes at a similar level were observed in the samples with coffee extract and cinnamon extract. As a result of accelerated aging, the MFR values of these materials increased by 0.36 g/10 min for coffee extract and 0.24 g/10 min for cinnamon extract. Unfortunately, it only applied to the lowest extract contents, i.e., 0.5 wt.%. Increasing the content of the extracts was disadvantageous considering the stability of the flow parameter. With the increase in the amount of coffee and cinnamon extracts in the material, the difference between the MFR values of the unaged sample and the aged sample increased significantly, reaching the maximum at 10 wt.% of the extracts. Therefore, for 10 wt.% of coffee extract, the difference between the unaged sample and the sample aged for 2160 h was 0.81 g/10 min and for 10 wt.% of the cinnamon extract was 1.45 g/10 min. Thus, coffee and cinnamon extracts with higher contents intensify the adverse effects occurring during aging, negatively affecting the rheological properties of the material. An interesting effect was observed for cocoa extract. In this case, the MFR value was most similar to unaged PCL, and the smallest changes in this value due to accelerated aging were obtained for the sample with the highest extract content, i.e., 10 wt.%.

The significant increase in MFR due to the aging of samples containing a large number of extracts can be explained by the fact that in the case of polyphenols, there is a certain limit amount above which they lose their anti-aging properties. In larger amounts, these compounds, instead of stopping the aging process, accelerate it significantly. As a result, we observe a significant increase in flow because of intensified degradation processes.

### 3.4. Tensile Tests

Based on the static tensile strength tests, the tensile strength (σ_M_) and tensile strain at break (ε_B_) were determined. The aging coefficients (K) of polymeric materials calculated from Equation (4) were also determined. The 2160 h aging time values were used as the values for “after aging”.

(4) $$K=\frac{{\left(\sigma_{M}\cdot \varepsilon_{B}\right)}_{\mathrm{after}\;\mathrm{aging}}}{{\left(\sigma_{M}\cdot \varepsilon_{B}\right)}_{\mathrm{befor}\;\mathrm{aging}}}$$

The results of static tensile strength tests on unaged and aged samples together with K values are presented in Table 3.

The value of σ_M_ of pure, unaged PCL was 45.8 MPa. When the reference anti-aging compound was added, the tensile strength of the unaged sample R increased by 3.6 MPa. As PCL is highly elastic at ambient temperature, the obtained deformations are large. The ε_B_ values of the unaged P and R samples were 1531% and 1463%, respectively.

Strengthening of the materials was also observed after adding the tested extracts to the matrix. Even a small content of all tested extracts caused a significant increase in the σ_M_ value. The σ_M_ values of samples containing 0.5 wt.% of coffee, cocoa, or cinnamon extract increased by 3.7; 4.5, and 5.2 MPa. Unfortunately, with higher contents of cocoa and cinnamon extracts, the tensile strength deteriorated. In the case of cocoa extract already at a content of 1 wt.%, the obtained values of σ_M_ were lower than the values of pure PCL and they decreased further along with the increase in the extract content. For cinnamon extract, a similar decrease in tensile strength was observed from 5 wt.% of extract. The exception was the coffee extract, for which the tensile strength was better than for pure PCL even at higher extract contents. The parameter ε_B_ of samples containing coffee extract was also at a similar level, where the obtained values ranged from 1473 to 1537; therefore, they were close to the value of the sample P. In the case of cocoa and cinnamon extracts, there was a decrease in the ε_B_ value from 1519 to 1266% for cocoa extract and from 1472 to 1182% for cinnamon extract with an increase in the extract content. The initial increase in strength is due to a slight increase in the crystallinity of the materials (which is confirmed by further DSC studies). The deterioration of mechanical properties at elevated levels of extracts may be related to the high content of the carrier introduced into the matrix together with the active substances of the extracts and the solubility of extracts. A detailed description of changes in mechanical properties is provided in the previous work [44].

**Figure 4:**
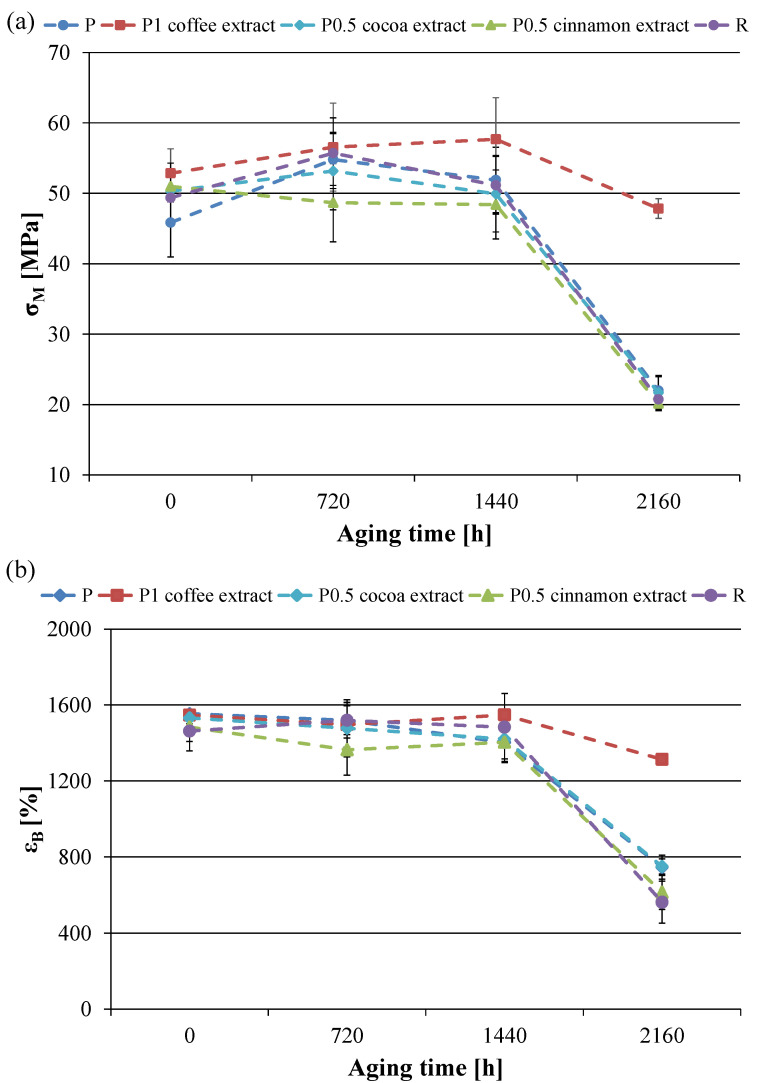
Values of (**a**) tensile strength (σ_M_) and (**b**) strain at break (ε_B_) of selected samples as a function of aging time.

As the best tensile properties were obtained for low extract contents, a more detailed analysis of the influence of accelerated aging on the mechanical parameters was carried out on these materials (Figure 4).

The tensile strength of sample P decreased significantly after 2160 h of accelerated aging. Initially, however, material strengthening was observed as the recorded value of σ_M_ after 720 h aging increased by 9.0 MPa. After this time, the tensile strength of the material deteriorated, with a large decrease in the value of σ_M_ by 23.8 MPa after 2160 h of aging. The initial strengthening of the material with a further decrease in tensile strength with increasing aging time was also observed for the R sample. The total decrease in the value of σ_M_ after 2160 h of aging for this material was 28.7 MPa. Thus, the reference anti-aging compound did not inhibit the adverse effect of accelerated aging on PCL tensile strength.

Cocoa and cinnamon extracts were characterized by a similar ineffectiveness. As in the case of samples P and R, for samples containing cocoa and cinnamon extracts, after 2160 h of accelerated aging there was a sharp drop in the value of σ_M_. For materials containing 0.5 wt.%. cocoa and cinnamon extracts, the σ_M_ values decreased by 28.6 MPa and 30.9 MPa, respectively, compared to the values of unaged samples. On the other hand, the coffee extract showed the ability to inhibit the decrease in tensile strength. This substance limited the decrease in tensile strength after 2160 h of accelerated aging observed in the case of other materials. The most stable parameters as a function of aging time were found in the material containing 1 wt.% of coffee extract where the difference between the values of the unaged and the aged samples was only 5.1 MPa. The difference was therefore much smaller than the differences observed in the other samples. It is worth noting that the positive effect of the coffee extract was maintained even at higher concentrations. The coffee extract positively influenced the stability of the tensile strength up to 5 wt.%. The coffee extract also had a positive effect on the recorded deformations of aged samples. Unlike other materials, the decrease in ε_B_ of a sample containing 0.5 wt.%. coffee extract after 2160 h of accelerated aging was much smaller. The ε_B_ value decreased from 1473 to 1299%, i.e., by 174 units, so the material was still very elastic. This decrease was much smaller than in the other samples, where the ε_B_ values dropped by 780–1455 units, and most of the samples became very brittle.

The initial increase in tensile strength observed after shorter aging times was associated with an increase in the degree of crystallinity of the materials. As demonstrated by the DSC tests presented below, the degree of crystallinity of the materials remained at a similar level despite the longer aging time; however, a decrease in tensile strength and strain at the break was observed. The decrease in mechanical properties is probably related to the degradation of the amorphous phase, which is more susceptible to UV radiation [48].

The aging coefficient characterizes the degree of material degradation as an effect of aging processes. A value of K close to zero indicates a high susceptibility to aging while values closer to one indicate high aging resistance [43]. Calculations of the K coefficient confirm that the coffee extract has the best anti-aging properties, as the obtained K values for most samples are exceedingly high. In the case of the remaining extracts, the calculated K values did not differ significantly from the values obtained for the P and R samples.

### 3.5. Impact Tensile Tests

The results of the impact strength (a_tU_) tests are presented in Table 4.

During the tensile impact test, only a few samples broke, while P and R, along with most materials containing coffee extract and samples with up to 3 wt.% of cocoa and cinnamon extracts, showed no signs of fracture. However, an observed decrease in the value of atU was noticed in samples with cocoa and cinnamon extract when the extract content exceeded 3 wt.%. The samples containing 10 wt.% mass of extracts exhibited the lowest values with a decrease of 38.2 or 48.2 kJ/m^2^, respectively, compared to samples containing 3 wt.% of extracts. The material containing 10 wt.% of coffee extract had the highest a_tU_ value among the samples that broke. The a_tU_ value was about twice that of samples with the same concentration of cocoa or cinnamon extracts. The negative effect on impact tensile strength during the tensile test may be due to the active substance carrier contained in the added extracts and the difference in the solubility of contained polyphenols. According to [36], the type and amount of polyphenols present in the polymer matrix determines how much of them can dissolve. Most of the time, polyphenols lose some of their solubility as their concentration rises. The matrix develops precipitation, which has a detrimental effect on the created materials’ mechanical properties.

The impact tensile strength of pure PCL did not change under the influence of accelerated aging up to 1440 h. Up to that aging time, the R samples did not break during the impact test. Only after 2160 h of accelerated aging did the material break, and the recorded value of a_tU_ was 353.2 kJ/m^2^. Furthermore, the material with the reference anti-aging compound did not break until 1440 h of accelerated aging. The rupture of the R sample took place after 2160 h of aging, and the obtained a_tU_ value was 325.3 kJ/m^2^, i.e., 27.9 kJ/m^2^ lower than the value of the aged P sample.

Again, the most favorable anti-aging effects were observed with lower extract contents. Materials containing 0.5 wt.% cocoa and cinnamon extracts under the influence of accelerated aging behaved similarly to pure PCL and the material containing the reference anti-aging compound. Up to 1440 h of aging the samples did not break. The a_tU_ values obtained after 2160 h of aging were 272.3 kJ/m^2^ for cocoa extract and 231.5 kJ/m^2^ for cinnamon extract. However, they were lower than the value obtained for the aged R sample. Therefore, it can be assumed that cocoa and cinnamon extracts showed lower anti-aging properties than the reference compound.

The coffee extract had better properties than the other extracts and the reference anti-aging compound. Even with 0.5 wt.% of coffee extract and after 2160 h of accelerated aging, the samples did not break during the tensile impact test. The positive effect of the coffee extract was maintained also at higher contents, as even samples containing 5 wt.% did not break after the longest accelerated aging time.

### 3.6. Thermogravimetric Analysis

**Figure 5:**
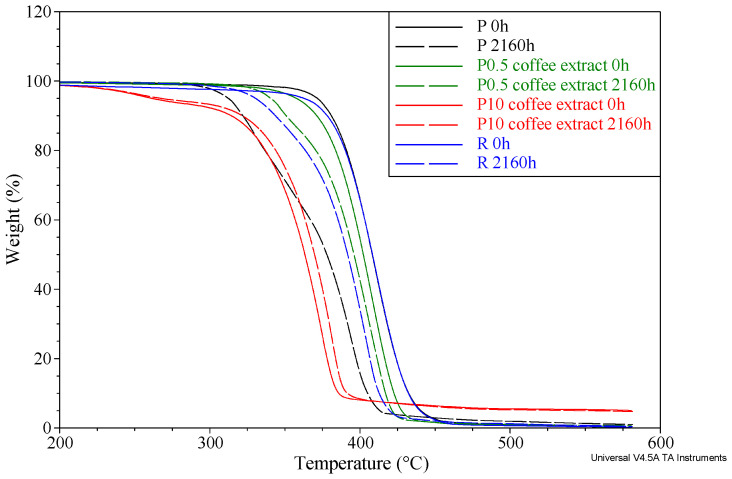
TG curves of selected samples before and after the aging process.

Figure 5 shows the thermogravimetric (TG) curves of selected samples before and after the aging process. In the TG analysis the temperature of the loss of 5% of the mass of the tested sample T_5%_ was determined. The T_5%_ value was taken as a parameter defining the beginning of thermal degradation of the material. The T_5%_ results of all samples are shown in Table 5.

When PCL is subjected to thermal degradation in an inert atmosphere, the polyester chains break down due to an ester pyrolysis reaction, which results in the release of CO_2_, H_2_O, and carboxylic acids. Through pyrolysis, the chains of macromolecules experience random cleavages [51]. The P sample has a T_5%_ value of 371.7 °C. Adding the reference anti-aging agent reduced the T_5%_ by 8.9 °C. The tested extracts also reduced the T_5%_ compared to the value obtained for the P and R samples. When 0.5% of coffee, cocoa, or cinnamon extracts were added to PCL, T_5%_ decreased by 14.0, 9.4, and 2.3 °C, respectively, compared to pure PCL. However, the most significant decrease was observed in samples containing 10% extracts, with T5% values being lower by 110.1 °C for coffee extract, 58.4 °C for cocoa extract, and 74.3 °C for cinnamon extract compared to sample P. The observed decrease in thermal resistance after adding the extracts may be caused by two factors. The first is the introduction to the matrix of compounds that are characterized by lower thermal resistance than the matrix material [52]. Secondly, the plasticizing effect of the extracts was visible in the MFR studies. According to reports [53], incorporating plasticizers into biopolyesters leads to a reduction in the thermal stability of the polymer.

**Figure 6:**
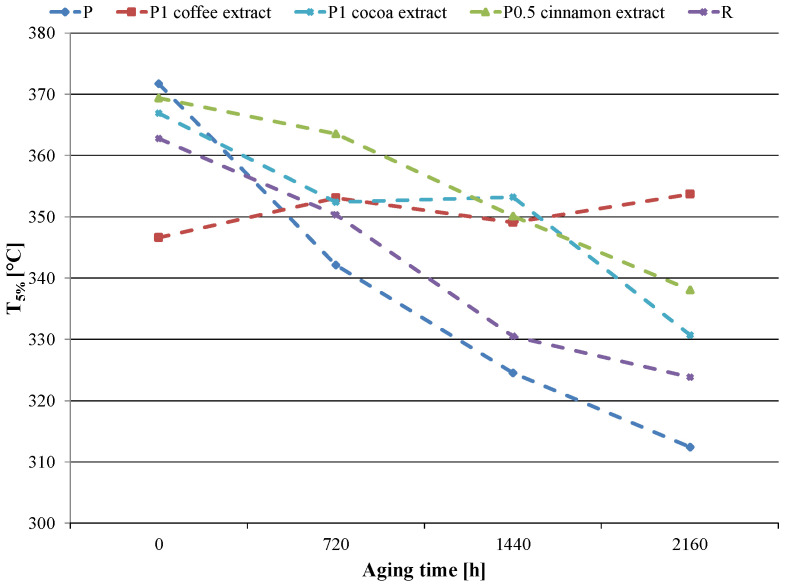
The temperature of 5% weight loss (T_5%_) values of selected samples as a function of aging time.

The thermal resistance of pure PCL decreased significantly with the progress of the accelerated aging time. The T_5%_ value of the P sample decreased approximately linearly with increasing aging time (Figure 6). The difference in T_5%_ between the sample aged for 2160 h and the unaged sample was 59.3 °C. By adding a reference anti-aging compound to the matrix, the material’s thermal resistance was enhanced. The T_5%_ value of the R sample aged for 2160 h was lower by 39.0 °C than the unaged sample, so the decrease was smaller than that observed for the P sample.

With low contents of extracts (up to 1% by weight), the proposed substances positively influenced the changes in thermal resistance as a function of aging time without causing too large of an initial decrease in thermal resistance. In the case of cocoa and cinnamon extracts, the behavior of samples containing these extracts under the influence of accelerated aging was similar to that of sample R. As the aging time progressed, a decrease in the value of T_5%_ was observed but the changes were smaller than in the case of sample P, which indicates better anti-aging properties of the extracts. The differences in T_5%_ between the samples aged for 2160 h and the unaged samples were 26.2 °C for 1 wt.% of cocoa extract and 31.3 °C for 0.5 wt% of cinnamon extract. In the case of coffee extract, the course of changes in thermal resistance as a function of aging time was different. Despite the initial decrease in T_5%_ after adding 1 wt.% of coffee extract, the value of this parameter did not change significantly due to accelerated aging. The T_5%_ after 2160 h of accelerated aging was practically at the same level as the value obtained for the unaged sample. Thus, it was the coffee extract that showed the best anti-aging properties, taking into account the effectiveness in limiting the decrease in thermal resistance.

### 3.7. Differential Scanning Calorimetry

The analysis of the DSC results focused on the first and second heating runs. Through the first heating, we determined how the selected thermal properties of the samples were affected by accelerated aging. The second heating, undertaken after removing the thermal history, helped to determine how accelerated aging affected the structure of the tested materials. Using the DSC test, the melting point (T_m1_ and T_m2_) and degree of crystallinity (X_c1_ and X_c2_) for each heating cycle were determined. Unfortunately, the DSC curves did not show the glass transition of PCL despite the suitable temperature range. The values of X_c_ were calculated based on Formula (1), assuming that the value of the enthalpy change of 100% crystalline PCL (Δ_Hm100%_) is 139.5 J/g [54].

(5) $$X_{c}=\left(\frac{{∆H}_{m}}{{∆H}_{m100\%}}\right)\cdot 100\%$$

**Figure 7:**
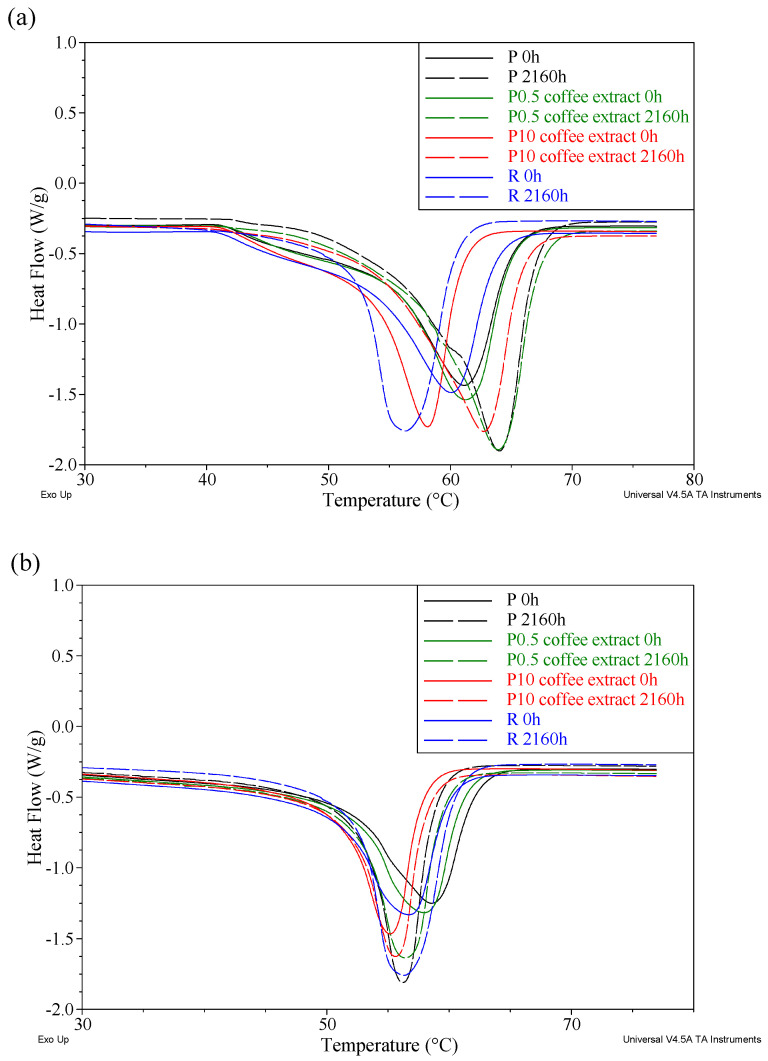
DSC curves of unaged and aged samples containing coffee extract; (**a**) first heat (**b**) second heat.

Examples of the curves of the first and second heating of selected unaged and aged samples are shown in Figure 7.

Based on the results of the first heating (Table 6), it can be concluded that the accelerated aging process did not significantly affect the phase changes of the tested materials. The melting point (T_m1_) of sample P was 61.2 °C with a degree of crystallinity (X_c1_) of 46.7%. The addition of the reference compound or the tested extracts to the matrix did not significantly affect the thermal parameters of unaged samples. Only a slight increase in the degree of crystallinity was observed for the samples containing the tested extracts, probably caused by the appearance of additional nucleation centers (active substance carrier particles).

As a result of the accelerated aging process, the T_m1_ value slightly increased, which was related to the increasing degree of crystallinity X_c1_ of the samples, which in practically all cases reached its maximum after 2160 h of aging. The observed increase in the degree of crystallinity with aging is a typical phenomenon accompanying the conditioning of polymeric materials for a long time at elevated temperatures, which is related to the ordering of the polymer chains.

By analyzing the results obtained based on the second heating (Table 7), i.e., when the influence of the accelerated aging process itself was eliminated, it was found that the melting point (T_m2_) of the unaged P sample decreased to 58.6 °C with a simultaneous decrease in the degree of crystallinity (X_c2_) to 42.8%. A similar dependence was observed for the unaged samples containing the tested anti-aging compounds, where a decrease in these two parameters was also observed. However, no major influence of the accelerated aging process on the melting point (T_m2_) and the degree of crystallinity (X_c2_) of the tested materials was noticed. The obtained differences between the samples aged for 2160 h and the aged samples were insignificant. This was applied to both pure PCL and materials containing the reference anti-aging compound as well as the tested extracts.

Contrary to the studies presented above, in the case of DSC studies, there was no clear relationship between the contents of individual extracts and the values of the recorded parameters of aged materials. Regardless of whether the material contained 0.5 or 10 wt.% of the extract, the values of the tested samples at individual stages of aging were similar.

## 4. Conclusions

The purpose of the study was to investigate the potential of utilizing coffee, cocoa, and cinnamon extracts as natural anti-aging agents in a biodegradable polymer known as polycaprolactone. The effectiveness of these extracts was evaluated through FTIR analysis and by observing changes in selected properties of polycaprolactone in relation to extract concentration and aging duration. The results were compared against those of a control sample containing a synthetic anti-aging compound, BHT.

The research suggests that the plant proposed extracts possess anti-aging properties. However, it is important to note that higher concentrations of the extracts can alter the properties of polycaprolactone, making it beneficial to use amounts below 1 wt.%. At this concentration, the extracts can effectively prevent the harmful effects of accelerated aging while maintaining the original properties of polycaprolactone-based materials. Additionally, using lower concentrations of the extracts can prevent the decrease in mechanical strength, thermal resistance, and increase in melt flow index during the aging process.

Coffee extract in an amount up to 1 wt.% turned out to be the best anti-aging compound, in most cases giving results better than the results obtained for the reference anti-aging compound. The material with coffee extract after 2160 h of aging was characterized by an acceptable increase in the melt rate by approx. 0.4 g/10 min (0.17 g/10 min in the case of the reference anti-aging compound), a decrease in tensile strength by approx. 6.0 MPa (28.6 MPa for the reference anti-aging compound), a decrease in the strain at fracture of approximately 200 units (901 units for the reference anti-aging compound), no breakage of the samples in the tensile impact test (the sample with the reference anti-aging compound cracked), and no loss of thermal stability (32.3 °C reduction in resistance with the reference anti-aging compound).

Based on the results obtained, plant extracts could be utilized in an industrial setting to produce eco-friendly packaging materials as an alternative to traditional polymer products.

## Figures and Tables

**Table 1:** Color test results.

	Extract Content [%wt]	Aging Time [h]								
		0			720			2160		
		L	ΔE	BI/YI *	L	ΔE	BI/YI *	L	ΔE	BI/YI *
P	-	91.67	-	3.37 *	91.52	3.37	13.07 *	92.65	14.35	27.79 *
Coffee extract	0.5	85.88	13.04	0.78	82.51	29.86	4.89	91.94	3.47	−0.34
	1	69.87	35.10	12.81	60.14	47.62	22.48	91.12	4.41	−0.39
	3	71.66	33.32	11.63	46.39	56.79	31.79	45.05	53.53	28.01
	5	62.45	37.98	12.11	42.76	56.28	29.44	49.63	47.99	24.16
	10	55.54	40.30	12.02	38.17	57.71	26.76	38.4	57.06	24.75
Cocoa extract	0.5	79.23	22.80	6.40	88.35	18.89	0.73	91.77	4.16	−0.37
	1	71.75	33.32	11.65	82.44	29.16	5.41	91.25	5.45	−0.17
	3	54.84	48.99	23.37	59.84	49.02	25.41	85.78	15.94	2.40
	5	44.65	54.35	29.49	53.16	51.71	29.45	67.58	40.92	17.10
	10	35.04	59.74	30.34	41.28	53.72	23.31	55.35	40.69	15.28
Cinnamon extract	0.5	83.18	17.73	3.33	89.34	18.14	0.26	90.92	6.66	0.06
	1	82.66	20.41	4.31	86.84	20.59	2.14	88.79	11.22	1.28
	3	57.61	48.48	22.27	79.09	31.45	8.37	81.59	24.90	6.67
	5	57.76	47.96	21.80	68.54	43.56	18.68	68.01	42.70	19.21
	10	46.8	53.46	28.32	47.9	53.49	31.68	61.58	45.74	24.13
R	-	92.31	0.64	8.59 *	90.91	6.31	17.13 *	92.64	18.13	32.61 *

* YI.

**Table 2:** The results of the melt flow rate (MFR) tests.

	Extract Content [%wt]	Aging Time [h]			
		0	720	1440	2160
		MFR [g/10 min]	MFR [g/10 min]	MFR [g/10 min]	MFR [g/10 min]
P	-	1.65 ± 0.07	1.83 ± 0.05	1.85 ± 0.06	1.89 ± 0.09
Coffee extract	0.5	1.76 ± 0.08	1.9 ± 0.08	1.98 ± 0.11	2.12 ± 0.15
	1	1.85 ± 0.03	1.92 ± 0.04	2.04 ± 0.04	2.27 ± 0.16
	3	1.84 ± 0.03	1.96 ± 0.03	2.14 ± 0.09	2.43 ± 0.18
	5	1.90 ± 0.03	1.96 ± 0.04	2.16 ± 0.15	2.46 ± 0.16
	10	1.96 ± 0.04	2.19 ± 0.084	2.84 ± 0.14	2.77 ± 0.15
Cocoa extract	0.5	2.08 ± 0.08	2.19 ± 0.08	2.59 ± 0.09	2.95 ± 0.05
	1	1.99 ± 0.03	2.33 ± 0.08	2.63 ± 0.11	2.99 ± 0.11
	3	2.05 ± 0.04	2.25 ± 0.12	2.50 ± 0.11	3.16 ± 0.08
	5	1.95 ± 0.05	2.03 ± 0.05	2.48 ± 0.11	2.87 ± 0.12
	10	1.71 ± 0.04	1.71 ± 0.03	1.96 ± 0.11	2.42 ± 0.15
Cinnamon extract	0.5	1.79 ± 0.02	1.8 ± 0.08	1.88 ± 0.06	2.03 ± 0.10
	1	1.68 ± 0.02	1.81 ± 0.05	1.85 ± 0.11	2.09 ± 0.06
	3	1.60 ± 0.03	1.77 ± 0.07	1.93 ± 0.09	2.22 ± 0.07
	5	1.76 ± 0.06	1.99 ± 0.04	2.15 ± 0.09	2.72 ± 0.07
	10	1.94 ± 0.04	2.07 ± 0.10	2.47 ± 0.12	3.39 ± 0.16
R	-	1.89 ± 0.03	1.95 ± 0.03	1.91 ± 0.05	2.06 ± 0.17

**Table 3:** Tensile test results.

	Extract Content [%wt]	Aging Time [h]								K
		0		720		1440		2160		
		σ_M_ [MPa]	ε_B_ [%]	σ_M_ [MPa]	ε_B_ [%]	σ_M_ [MPa]	ε_B_ [%]	σ_M_ [MPa]	ε_B_ [%]	
P	-	45.8 ± 4.9	1531 ± 29	54.8 ± 3.7	1494 ± 62	51.9 ± 4.7	1393 ± 87	22.0 ± 2.0	745 ± 65	0.23
Coffee extract	0.5	49.5 ± 5.7	1473 ± 132	53.5 ± 8.2	1445 ± 157	53.0 ± 5.1	1464 ± 127	42.2 ± 8.1	1299 ± 17	0.75
	1	52.9 ± 3.5	1537 ± 24	56.6 ± 6.2	1486 ± 121	57.7 ± 5.9	1527 ± 114	47.8 ± 1.4	1310 ± 6	0.77
	3	47.9 ± 4.6	1484 ± 109	50.5 ± 4.8	1372 ± 93	54.0 ± 3.7	1454 ± 90	46.6 ± 3.0	1279 ± 52	0.84
	5	51.5 ± 2.6	1522 ± 76	54.2 ± 4.4	1423 ± 102	51.2 ± 9.0	1368 ± 196	41.1 ± 10.3	1287 ± 39	0.67
	10	48.3 ± 4.3	1480 ± 47	41.5 ± 5.0	1292 ± 161	28.0 ± 2.5	892 ± 280	29.8 ± 3.6	911 ± 197	0.38
Cocoa extract	0.5	50.3 ± 4.0	1519 ± 35	53.2 ± 5.5	1466 ± 146	49.9 ± 5.4	1405 ± 114	21.7 ± 2.5	739 ± 38	0.21
	1	43.5 ± 4.7	1465 ± 125	49.1 ± 2.8	1411 ± 75	48.1 ± 3.5	1404 ± 78	22.4 ± 3.5	729 ± 38	0.26
	3	44.5 ± 2.7	1445 ± 67	42.7 ± 1.6	1299 ± 56	34.0 ± 8.3	1046 ± 309	21.3 ± 2.0	724 ± 63	0.24
	5	42.4 ± 3.8	1323 ± 68	38.9 ± 1.1	1226 ± 49	34.7 ± 1.9	1181 ± 41	20.8 ± 3.0	715 ± 20	0.27
	10	33.8 ± 2.1	1266 ± 15	35.9 ± 2.4	1181 ± 56	33.0 ± 2.1	1112 ± 41	20.8 ± 5.3	717 ± 26	0.35
Cinnamon extract	0.5	51.0 ± 2.2	1472 ± 83	48.7 ± 5.6	1351 ± 126	48.4 ± 4.9	1384 ± 118	20.1 ± 1.0	614 ± 89	0.16
	1	50.6 ± 7.0	1494 ± 47	48.3 ± 1.8	1405 ± 81	44.5 ± 2.9	1362 ± 84	19.3 ± 0.5	602 ± 86	0.15
	3	59.8 ± 7.4	1349 ± 75	41.3 ± 2.2	1254 ± 35	35.7 ± 6.4	1205 ± 108	22.0 ± 5.5	584 ± 211	0.16
	5	43.5 ± 5.4	1251 ± 58	39.5 ± 2.0	1210 ± 42	35.0 ± 3.1	1194 ± 49	22.3 ± 6.2	906 ± 179	0.37
	10	28.3 ± 3.0	1182 ± 92	29.8 ± 2.2	1080 ± 46	26.0 ± 5.1	1018 ± 135	16.6 ± 0.9	29 ± 9	0.01
R	-	49.4 ± 3.9	1463 ± 104	55.7 ± 5.0	1510 ± 91	51.2 ± 4.1	1467 ± 76	20.7 ± 1.3	562 ± 110	0.16

**Table 4:** Impact tensile strength results (a_tU_); NB—did not break.

	Extract Content [%wt]	Aging Time [h]			
		0	720	1440	2160
		a_tU_ [kJ/m^2^]	a_tU_ [kJ/m^2^]	a_tU_ [kJ/m^2^]	a_tU_ [kJ/m^2^]
P	-	NB	NB	NB	353.2 ± 34.2
Coffee extract	0.5	NB	NB	NB	NB
	1	NB	NB	NB	NB
	3	NB	NB	NB	NB
	5	NB	NB	NB	NB
	10	268.2 ± 28.4	282.2 ± 21.4	172.9 ± 26.1	167.2 ± 26.6
Cocoa extract	0.5	NB	NB	NB	272.3 ± 23.0
	1	NB	NB	NB	249.6 ± 13.7
	3	187.7 ± 26.1	290.6 ± 55.2	221.3 ± 19.1	224.4 ± 18.0
	5	187.6 ± 32.6	202.9 ± 18.6	202.5 ± 23.4	192.6 ± 17.2
	10	149.5 ± 27.6	192.1 ± 19.3	180.3 ± 24.9	184.6 ± 12.2
Cinnamon extract	0.5	NB	NB	NB	231.5 ± 31.0
	1	NB	NB	316.8 ± 83.0	237.6 ± 15.3
	3	182.8 ± 28.0	223.1 ± 39.4	226.9 ± 32.7	195.3 ± 19.4
	5	173.5 ± 20.7	191.5 ± 28.8	199.5 ± 26.6	189.6 ± 13.3
	10	134.6 ± 16.1	138.6 ± 13.7	163.0 ± 9.3	160.4 ± 8.1
R	-	NB	NB	NB	325.3 ± 55.5

**Table 5:** Results of thermogravimetric tests.

	Extract Content [%wt]	Aging Time [h]			
		0	720	1440	2160
		T_5%_ [°C]	T_5%_ [°C]	T_5%_ [°C]	T_5%_ [°C]
P	-	371.7	342.1	324.5	312.4
Coffee extract	0.5	357.7	349.1	357.4	340.6
	1	346.6	353.1	349.1	353.7
	3	339.0	330.9	342.4	342.8
	5	326.1	299.7	312.9	321.8
	10	261.6	305.3	303.4	267.4
Cocoa extract	0.5	362.4	366.6	334.0	336.2
	1	366.9	352.4	353.2	330.7
	3	357.0	357.4	333.1	344.2
	5	346.3	338.9	338.6	331.4
	10	313.3	317.1	297.8	307.8
Cinnamon extract	0.5	369.4	363.5	350.1	338.1
	1	368.3	353.1	339.2	341.1
	3	362.2	346.1	346.6	334.0
	5	353.8	346.2	359.2	326.5
	10	350.3	315.8	310.7	297.4
R	-	362.8	350.3	330.5	323.8

**Table 6:** Results of the first heating scan.

	Extract Content [%wt]	Aging Time [h]							
		0		720		1440		2160	
		T_m1_ [°C]	X_c1_ [%]	T_m1_ [°C]	X_c1_ [%]	T_m1_ [°C]	X_c1_ [%]	T_m1_ [°C]	X_c1_ [%]
P	-	61.2	46.7	62.8	54.2	62.5	58.5	64.0	56.8
Coffee extract	0.5	61.2	48.2	62.9	55.2	62.6	55.7	63.9	57.2
	1	61.0	49.0	61.8	53.8	62.7	55.3	64.6	55.5
	3	60.5	47.8	63.8	51.3	63.1	54.7	63.3	54.1
	5	62.5	48.3	63.1	51.4	62.1	53.9	63.5	53.7
	10	58.2	43.3	63.1	49.7	62.9	52.4	62.8	50.9
Cocoa extract	0.5	60.3	49.0	62.5	55.5	62.9	56.1	64.4	58.6
	1	61.3	49.1	62.8	53.8	64.4	57.0	64.0	57.7
	3	59.5	48.2	65.1	52.2	63.8	53.8	63.4	55.8
	5	59.1	48.7	62.7	53.4	63.0	52.4	63.3	54.9
	10	59.6	44.4	62.7	50.8	63.5	51.2	62.7	53.0
Cinnamon extract	0.5	59.2	47.7	63.6	52.8	62.9	58.0	63.9	58.5
	1	59.5	47.7	63.2	53.4	64.4	56.7	64.2	58.7
	3	62.6	49.3	63.4	51.9	63.8	55.5	64.2	58.1
	5	58.5	46.1	62.2	51.4	63.0	52.7	63.8	57.3
	10	60.5	46.6	63.2	51.3	63.5	55.7	62.7	57.5
R	-	60.1	46.1	62.0	55.3	62.6	58.5	64.3	65.2

**Table 7:** Results of the second heating scan.

	Extract Content [%wt]	Aging Time [h]							
		0		720		1440		2160	
		T_m2_ [°C]	X_c2_ [%]	T_m2_ [°C]	X_c2_ [%]	T_m2_ [°C]	X_c2_ [%]	T_m2_ [°C]	X_c2_ [%]
P	-	58.6	42.8	56.5	44.5	55.8	47.3	56.2	50.5
Coffee extract	0.5	57.9	44.3	56.1	45.0	56.2	45.4	56.4	44.1
	1	58.4	45.4	56.3	44.0	55.9	47.0	56.9	44.3
	3	57.0	47.1	57.4	42.6	56.5	46.1	56.1	43.5
	5	57.3	45.7	57.3	42.9	56.3	46.3	56.4	44.8
	10	55.2	42.7	57.2	41.6	56.2	44.5	55.6	41.0
Cocoa extract	0.5	56.6	44.6	56.1	44.1	57.0	47.4	56.5	45.6
	1	58.3	47.3	56.3	45.5	56.3	45.3	56.5	43.4
	3	55.8	49.6	58.3	43.9	55.9	45.4	56.2	46.2
	5	56.1	46.0	56.3	43.8	57.4	42.6	56.3	43.7
	10	56.8	43.0	56.7	43.3	55.9	42.2	56.0	39.4
Cinnamon extract	0.5	56.5	46.1	56.7	46.8	56.0	46.3	56.9	43.8
	1	56.3	47.5	56.4	43.5	56.8	44.9	56.3	47.2
	3	58.2	48.7	56.7	43.8	53.3	42.9	56.2	46.4
	5	56.7	45.6	56.2	40.7	56.2	44.4	55.9	44.1
	10	55.5	42.5	56.9	41.7	56.5	45.4	55.6	46.7
R	-	56.7	48.2	56.1	46.0	56.3	46.4	56.4	52.6

## Data Availability

Data are contained within the article.
